# Papillary renal neoplasm with reverse polarity is biologically and clinically distinct from eosinophilic papillary renal cell carcinoma

**DOI:** 10.1111/pin.13417

**Published:** 2024-03-08

**Authors:** Vincent Francis Castillo, Kiril Trpkov, Theodorus Van der Kwast, Fabio Rotondo, Malek Hamdani, Rola Saleeb

**Affiliations:** ^1^ Department of Laboratory Medicine and Pathobiology University of Toronto Toronto Ontario Canada; ^2^ Li Ka Shing Knowledge Institute St. Michael's Hospital Toronto Ontario Canada; ^3^ Department of Pathology and Laboratory Medicine Alberta Precision Laboratories and University of Calgary Calgary Alberta Canada; ^4^ Division of Pathology University Health Network Toronto Ontario Canada; ^5^ Department of Laboratory Medicine Unity Health Toronto Toronto Ontario Canada

**Keywords:** ABCC2, papillary renal cell carcinoma, papillary renal neoplasm with reverse polarity, prognosis

## Abstract

Papillary renal neoplasm with reverse polarity (PRNRP) is a recently described indolent entity with distinct features and its recognition from other oncocytic/eosinophilic papillary renal cell carcinoma (ePRCC) has important prognostic implications. ABCC2, a renal drug transporter, is overexpressed in aggressive PRCCs. In this study, we compared the clinicopathological parameters and the biological ABCC2 expression between PRNRP and ePRCC. PRNRP (*n* = 8) and ePRCC (*n* = 21) cases were selected from resection specimens and corresponding clinicopathological data were collected. ABCC2 immunohistochemical (IHC) staining was performed and ABCC2 staining patterns were classified as negative, cytoplasmic, and brush‐border. RNA in‐situ hybridization (ISH) was used to assess ABCC2 transcript levels. All eight PRNRP cases had weak cytoplasmic ABCC2 IHC reactivity; however, they showed no detectable ABCC2 transcripts on RNA ISH. In comparison, 76% (16/21) of ePRCCs showed ABCC2 IHC brush‐border expression and significantly higher ABCC2 RNA ISH transcript levels (*p* < 0.001). Additionally, the ePRCC group showed a significantly larger tumor size (*p* = 0.004), higher WHO/ISUP grade (*p* < 0.001), and stage (*p* = 0.044). None of the PRNRP cases showed disease progression, while 9.5% (2/21) ePRCCs had disease progression. PRNRP is clinically and biologically distinct from ePRCC. Hence, it is crucial to differentiate between these two entities, particularly in needle core biopsies.

**Abbreviations**
ABCC2: ATP binding cassette subfamily C member 2
ePRCC: eosinophilic papillary renal cell carcinoma
IHC: immunohistochemistry
ISH: in‐situ hybridization
ISUP: International Society of Urological Pathology
PRNRP: papillary renal neoplasm with reverse polarity
WHO: World Health Organization

## INTRODUCTION

In the recent WHO 2022 classification, papillary renal neoplasm with reverse polarity (PRNRP) has been recognized as one of the histological patterns of papillary renal cell carcinoma (PRCC).^1^ It comprises about 4% of PRCC, it has a specific low‐grade oncocytic morphology with a linear reverse arrangement of the nuclei, and it has a characteristic immunohistochemical (IHC) profile with GATA3 positivity and weak vimentin and racemase staining.^1^, ^2^, ^3^ PRNRP also commonly harbors specific *KRAS* mutations in exon 2, codon 12.^4^

The evidence gathered to date indicates that PRNRP has an indolent clinical course. Most of the reported cases are pT1a stage and often they are smaller in size, below the papillary adenoma size cutoff of 1.5 cm.^3^, ^4^ To our knowledge, there are no reported cases of disease progression, documented as recurrence or metastasis.^2^, ^3^ On the other hand, PRCCs with eosinophilic morphology were historically grouped within the high‐grade PRCC category, as part of the traditional PRCC type 2.^1^ Thus, the distinction of PRNRP from other eosinophilic PRCCs (ePRCC) has important prognostic implications.

ATP binding cassette subfamily C member 2 (ABCC2), a renal transporter protein, is highly expressed in PRCCs with more aggressive behavior, both at the transcriptomic and proteomic levels.^5^, ^6^, ^7^ Previous studies have assessed the prognostic value of ABCC2 as an IHC biomarker and have found that the pattern of prominent brush‐border reactivity was associated with higher ABCC2 transcript levels, as well as worse PRCC prognosis.^6^, ^7^ In this study, we aimed to compare the clinicopathological features, as well as the transcriptomic and proteomic expression of ABCC2 between PRNRP and ePRCC groups.

## MATERIALS AND METHODS

All cases were selected from nephrectomy resection specimens with formalin‐fixed, paraffin‐embedded tissue from four different institutions (Ontario Tumor Bank, Unity Health Toronto/St. Michael's Hospital, Unity Health Toronto/St. Joseph's Hospital, and Rockyview General Hospital/University of Calgary). A total of 177 PRCCs were reviewed by two pathologists (RS and VC). The final selected cohort included 29 cases. Eight cases were confirmed to be PRNRP based on specific morphology and IHC profile (GATA3, AMACR, and vimentin). Cases were selected as ePRCC if they had diffuse papillary morphology with abundant eosinophilic cytoplasm, and after exclusion of possible mimickers such as *TFE3*‐rearranged and *TFEB*‐altered RCC and fumarate hydratase‐deficient RCC using an appropriate IHC workup. Corresponding clinicopathological data and follow‐up information were also collected. This study received an approval from the Institutional Review Board at St. Michael's Hospital, Toronto, Ontario, Canada (20‐254), and was conducted in accordance with the Helsinki Declaration.

We performed an IHC staining with ABCC2 antibody (ABCAM, cat. no. ab187644, clone EPR10997(2), dilution 1:100).^6^ Cases were scored based on the IHC staining pattern as: negative, cytoplasmic, and brush‐border positive (also subdivided into <50% and ≥50%), as reported previously.^7^ RNA in‐situ hybridization (ISH) with ABCC2 transcript probe (ACDBio, cat. no. 1140921‐C1) was performed using the RNAscope® 2.5 HD assay.^8^ A semi‐quantitative scoring system (0–4) was adopted to assess ABCC2 transcript levels.^9^ Both ABCC2 IHC and RNA ISH were independently evaluated by two pathologists (RS and VC), and in cases of discrepancy, a final consensus was assigned after a joint review.

The clinicopathological features and the ABCC2 expression were compared using Fischer's exact test and *T* test and a *p* value of <0.05 was considered significant. Statistical analyses were performed using GraphPad Prism (Version 9.0).

## RESULTS

Clinicopathological data are summarized in Table 1. All PRNRPs had low‐grade (WHO/ISUP grades 1 and 2) morphology and all were pT1a stage. In contrast, ePRCCs were characterized by significantly larger tumor size (*p* = 0.004), higher WHO/ISUP grade (*p* < 0.001), and higher stage (*p* = 0.044) (Table 1). None of the PRNRP cases showed disease recurrence, while disease progression was documented in two ePRCC cases (9.5%).

**Table 1:** Comparison of the clinicopathological characteristics and ABCC2 expression between PRNRP and eosinophilic PRCC.

	**PRNRP (*n*** = **8)**	**Eosinophilic PRCC (*n*** = **21)**	***p* Value**
Age, mean (range), years	64 (51–79)	62 (35–83)	
Sex (M:F ratio)	1:1	3:1	
Tumor laterality (R:L ratio)	0.6:1	1.1:1	
Tumor size, mean (range), cm	1.3 (0.5–3.1)	5.9 (2.3–14)	***p*** = **0.004**
WHO/ISUP grade			***p*** < **0.001**
1–2 (low)	8	4	
3–4 (high)	0	17	
Pathological tumor stage			***p*** = **0.038**
pT1	8	12	
pT2	0	4	
pT3	0	5	
pT4	0	0	
Stage (AJCC 8th edition)			***p*** = **0.044**
I	8	12	
II	0	3	
III	0	4	
IV	0	2	
ABCC2 IHC pattern			***p*** < **0.001**
Negative	0	2	
Cytoplasmic	8	3	
Brush border <50%	0	7	
Brush border ≥50%	0	9	
ABCC2 RNA ISH score			***p*** < **0.001**
0	8	3	
1	0	1	
2	0	6	
3	0	3	
4	0	8	
Follow‐up, median (range), months	24 (5–124)	47 (1–209)	
Disease status			
No evidence of disease	6^a^	16	
Alive with disease	0	1 (recurrence)	
Dead of disease	0	1 (metastasis)	
Dead of other cause	0	3	

Abbreviations: ABCC2, ATP binding cassette subfamily C member 2; IHC, immunohistochemistry; ISH, in‐situ hybridization; ISUP, International Society of Urological Pathology; PRCC, papillary renal cell carcinoma; PRNRP, papillary renal neoplasm with reverse polarity; WHO, World Health Organization.

^a^ Follow‐up data not available for two PRNRPs.

**Figure 1:**
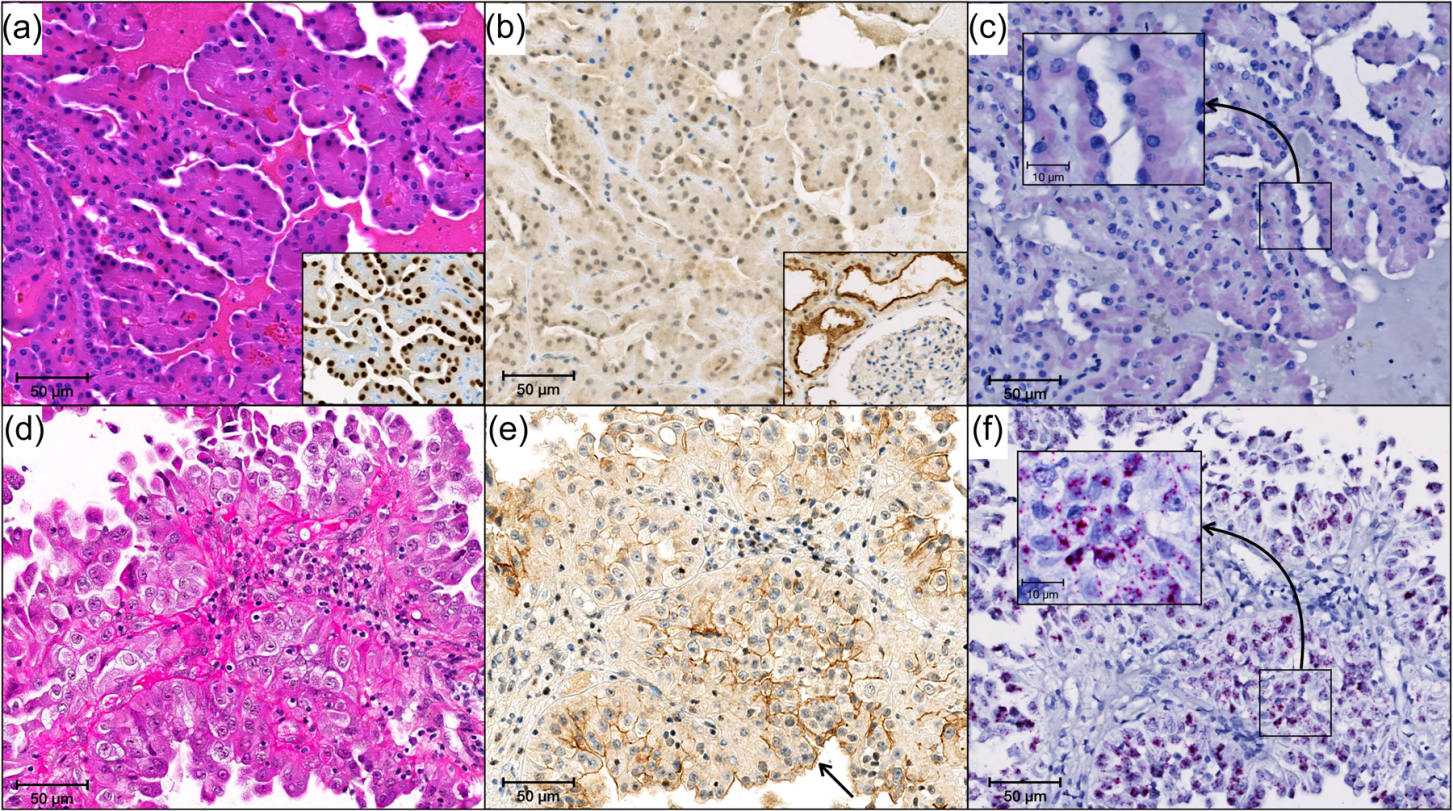
(a) Representative H&E of PRNRP with strong, diffuse GATA3 (inset); (b) PRNRP showing weak cytoplasmic ABCC2 staining (inset: proximal renal tubules as internal positive control and glomeruli as internal negative control); (c) PRNRP with RNA ISH score 0 (inset: higher magnification); (d–f) ePRCC with ABCC2 brush‐border ≥50% (brush‐border immunoreactivity indicated by an arrow) and RNA ISH score 4 (inset: higher magnification). ABCC2, ATP binding cassette subfamily C member 2; ePRCC, eosinophilic papillary renal cell carcinoma; H&E, hematoxylin and eosin; ISH, in‐situ hybridization; PRNRP, papillary renal neoplasm with reverse polarity.

Weak cytoplasmic staining for ABCC2 IHC was noted in all PRNRP cases; however, none of the PRNRPs showed any recognizable brush‐border reactivity. None of the PRNRP cases as well had any detectable ABCC2 transcript on RNA ISH (all cases had a score of 0) (Figure 1a–c). In comparison, the ePRCCs group showed that 76% (16/21) cases demonstrated positive ABCC2 IHC brush‐border reactivity, along with significantly higher ABCC2 transcript levels by RNA ISH (*p* < 0.001) (Figure 1d–f).

## DISCUSSION

Historically, low‐grade oncocytic papillary neoplasms were recognized as potentially more indolent and were referred to as “oncocytic” PRCCs.^10^, ^11^, ^12^ However, older studies also included a broader spectrum of PRCCs with eosinophilic morphology. Saleeb et al. described a subgroup of 6 PRCCs labeled “PRCC type 4” that had a low‐grade oncocytic morphology and a linear, apical arrangement of the nuclei. These cases were also exclusively GATA3 positive.^13^ Al‐Obaidy et al. further characterized examples of this entity in multiple studies and labeled it as PRNRP,^2^ which is the currently adopted nomenclature for this entity. To date, more than 100 PRNRP cases have been reported, and to the best of our knowledge, there are no known cases with documented disease progression or metastasis.^2^, ^3^, ^4^, ^13^, ^14^, ^15^, ^16^, ^17^, ^18^ Similar to the previous reports, we have also found that PRNRPs had significantly smaller tumor size, lower WHO/ISUP grade, and lower stage than ePRCCs. Additionally, no cases in our cohort were associated with recurrence or metastasis, unlike ePRCCs, which had 9.5% of cases showing progression. In fact, ePRCCs showed clinical features suggestive of a more aggressive PRCC phenotype.

All PRNRP cases had little to no ABCC2 RNA and protein expression, as evidenced by the negative RNA ISH assay and the absence of any brush‐border immunoreactivity on IHC, consistent with our previous findings.^7^ In contrast, ePRCCs had significantly higher ABCC2 mRNA levels as well as frequent ABCC2 IHC brush‐border reactivity pattern, also in keeping with our previous report documenting an association of brush‐border IHC reactivity with increased ABCC2 transcript levels.^7^ ABCC2 is a renal epithelial transmembrane transporter protein that is reported to be associated with an aggressive phenotype of PRCCs.^6^, ^7^ In our previous study, we found that ABCC2 brush‐border reactivity pattern (both <50% and ≥50%), was consistently associated with a significant increase in the ABCC2 transcript levels. In contrast, the weak cytoplasmic reactivity pattern (blush) was not associated with any significant increase in ABCC2 gene expression.^7^ ABCC2 is known to be implicated in chemotherapy resistance, but it is also thought to contribute to cancer aggressiveness beyond its potential role in treatment resistance.^17^ There is a growing body of evidence linking the increased ABCC2 expression with an aggressive PRCC biology, as documented for the ePRCC group in the current study. Thus, the lack of ABCC2 expression in PRNRP would be in keeping with the indolent nature of this entity. Of note, due to the limited number of PRNRP cases in this study, the findings require validation with a large cohort.

To date, there is no consensus regarding the clinical management of PRNRP. There is currently a clinical preference toward watchful waiting or active surveillance of small renal masses, particularly those considered benign or low malignant potential.^19^ Given the morphological similarities between PRNRP and ePRCC, it is crucial to distinguish between them, particularly on a limited needle core biopsy. Thus, a diagnosis of PRNRP on biopsy can guide the clinical management toward a conservative approach.^19^ On the other hand, ePRCC should benefit from a definitive surgical management.

## AUTHOR CONTRIBUTIONS

Rola Saleeb conceptualized and designed the study. Rola Saleeb and Kiril Trpkov provided sample materials. Rola Saleeb and Vincent Francis Castillo conducted data analysis and interpretation. Vincent Francis Castillo performed the statistical analysis. Vincent Francis Castillo drafted the manuscript. Vincent Francis Castillo, Kiril Trpkov, Theodorus Van der Kwast, and Rola Saleeb revised the manuscript. Fabio Rotondo and Malek Hamdani provided technical support. All authors listed read and approved the final paper.

## CONFLICT OF INTEREST STATEMENT

The authors declare no conflict of interest.
